# Daily variation in the prokaryotic community during a spring bloom in shelf waters of the East China Sea

**DOI:** 10.1093/femsec/fiy134

**Published:** 2018-07-13

**Authors:** Dong Han Choi, Sung Min An, Eun Chan Yang, Howon Lee, JaeSeol Shim, JinYong Jeong, Jae Hoon Noh

**Affiliations:** 1 Marine Ecosystem and Biological Research Center, Korea Institute of Ocean Science and Technology, Haeyang-ro, Yeongdo-gu, Busan 49111, Republic of Korea; 2 Department of Marine Biology, Korea University of Science and Technology, Gajeong-ro, Yuseong-gu, Daejeon 34113, Republic of Korea; 3 Operational Oceanography Research Center, Korea Institute of Ocean Science and Technology, Haeyang-ro, Yeongdo-gu, Busan 49111, Republic of Korea

**Keywords:** community composition, East China Sea, Ieodo Ocean Research Station, prokaryotes, shelf waters, spring bloom

## Abstract

To understand prokaryotic responses during a spring bloom in offshore shelf waters, prokaryotic parameters were measured daily at a station located in the middle of the East China Sea over a six-week period from March 25 to May 19. The site experienced a phytoplankton bloom in late April, triggering changes in prokaryotic abundance and production after a lag of approximately one week. Before the bloom, changes in prokaryotic composition were small. Both during the bloom and in the post-bloom period, successive changes among bacterial groups were apparent. A SAR11 group became more dominant during the bloom period, and diverse groups belonging to the *Flavobacteriia* occurred dominantly during both the bloom and post-bloom periods. However, bacterial community changes at the species level during the bloom and post-bloom periods occurred rapidly in a time scale of a few days. Especially, NS5, NS4 and *Formosa* bacteria belonging to *Flavobacteriia* and bacteria belonging to *Halieaceae* and *Arenicellaceae* families of *Gammaproteobacteria* showed a successive pattern with large short-term variation during the period. The changes in prokaryotic composition were found to be related to phytoplankton biomass and composition, as well as seawater temperature and variations in nutrients.

## INTRODUCTION

Algal blooms typically develop in the spring in temperate mid-latitude seas and are mainly triggered by a combination of higher light intensity/duration, nutrient levels, sea surface temperature and water column stratification (Smetacek and Cloern 2008; Taylor and Ferrari 2011). These environmental changes influence subsequent autotrophic and heterotrophic microbial growth, and result in changes in nutrients and organic matter conditions during the bloom. In a variety of aquatic systems, the organic matter produced by phytoplankton is a major factor regulating bacterial growth (Ducklow and Kirchman 1983; Cole, Findlay and Pace 1988; Ducklow *et al*. 1993). Further, development of distinct bacterial populations has been reported during phytoplankton blooms (Kerkhof *et al*. 1999; Riemann, Steward and Azam 2000; Yager *et al*. 2001). Although uncoupling between primary production and bacterial production has been reported (Billen and Fontigny 1987; Weisse and Scheffel-Moser 1991; Cho *et al*. 1994), bacterial biomass, production and composition should vary dynamically throughout a bloom. In coastal areas, diatoms and dinoflagellates are the main contributors to spring blooms (Kristiansen, Farbrot and Naustvoll 2001; Teeling *et al*. 2012; Needham and Fuhrman 2016). Accordingly, the community of prokaryotes, protists and viruses, which are closely linked to phytoplankton, show successive trends (Riemann, Steward and Azam 2000; Castberg *et al*. 2001; Yager *et al*. 2001; Larsen *et al*. 2004).

Different types of phytoplankton produce different kinds of organic matter, which are consumed by heterotrophic prokaryotes (Buchan *et al*. 2014; Xing *et al*. 2015). The responses of bacterial communities to algal blooms have been relatively well-studied in inshore coastal waters (Riemann, Steward and Azam 2000; Teeling *et al*. 2012; Yang *et al*. 2015; Sison-Mangus *et al*. 2016; Teeling *et al*. 2016). Algal blooms stimulate bacterial growth and thus cause a steep increase in bacterial abundance and production. Concurrently, bacterial composition also changes along bloom phases, as several genera belonging to Roseobacter, *Flavobacteriia* and *Gammaproteobacteria* were shown to consecutively dominate during spring bloom events (Riemann, Steward and Azam 2000; Teeling *et al*. 2016). Although the amount and chemical nature of extracellular dissolved organic matter (DOM) released depends on physiological states as well as diverse environmental conditions (Myklestad 1995; Meon and Kirchman 2001; Wetz and Wheeler 2007; Romera-Castillo *et al*. 2010), phytoplankton community are one of the most important factors in determining the DOM composition of seawater (Becker *et al*. 2014). Therefore, algal composition and succession are the most important factors in determining the subsequent responses of prokaryotes during blooms.

Using high throughput sequencing techniques, recent time-series studies on bacterial community composition changes during a spring bloom were conducted with high resolution over short intervals of a few days (Teeling *et al*. 2012; Needham and Fuhrman 2016; Teeling *et al*. 2016). However, time-series studies with short time intervals have rarely been conducted in offshore and shelf waters, likely due to the limited accessibility of the study area. Although satellite images have been used to find algal blooms in shelf waters (Maynard and Clark 1987; Thomas, Townsend and Weatherbee 2003; Park *et al*. 2014), the daily dynamics of microbial communities during offshore blooms are not yet well-resolved.

In this study in a central area of the East China Sea, daily observations of phytoplankton and prokaryote variables (biomass, productivity and community composition) were conducted for nearly two months to understand prokaryotic population responses to offshore algal blooms with high resolution on a one-day time scale.

## MATERIALS AND METHODS

### Study site and sampling

**Figure 1.**
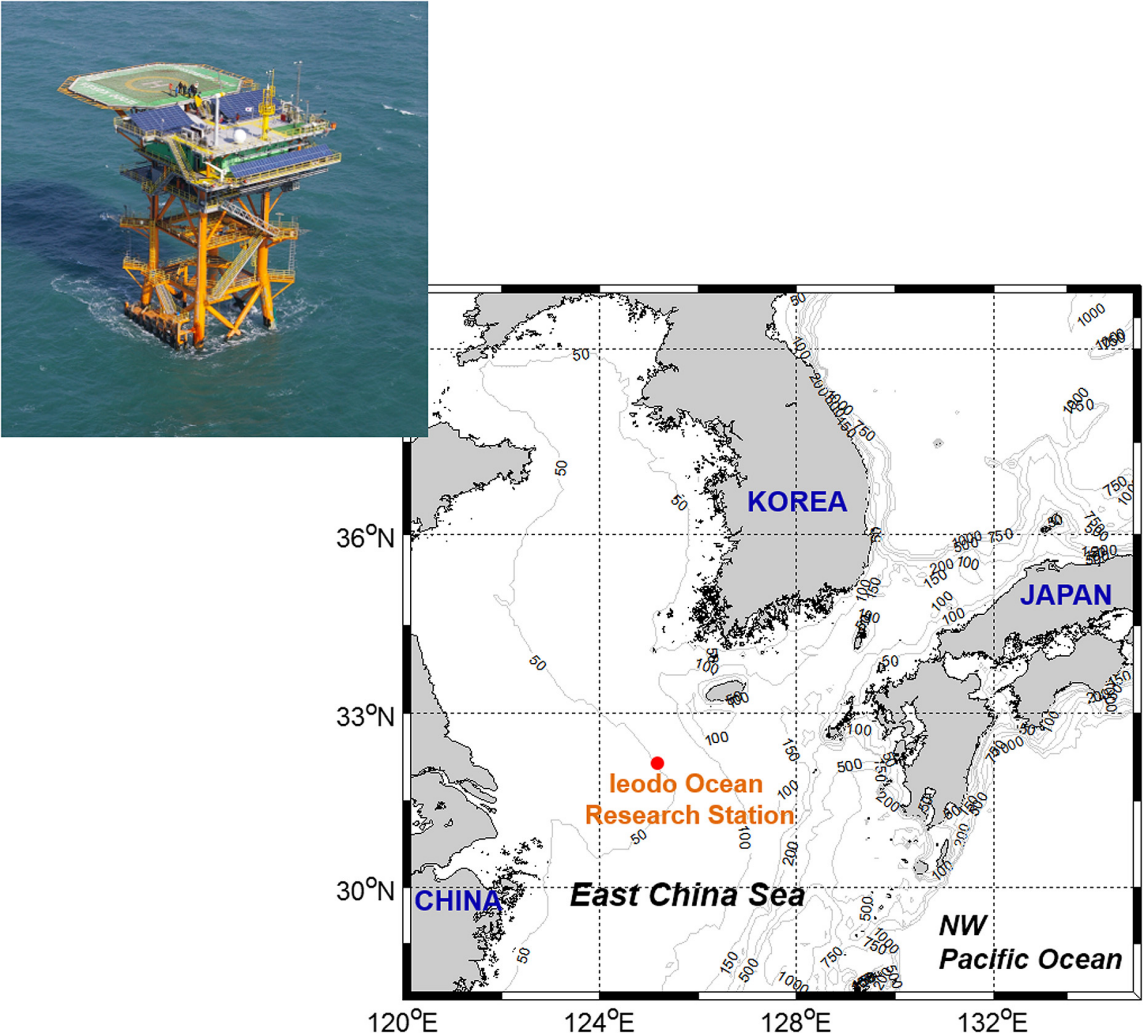
Map showing the study location, with a picture of the Ieodo Ocean Research Station.

This study was conducted on an oceanic and meteorological observation platform, near the middle of the East China Sea (Ieodo Ocean Research Station, IORS; 32°07’22’N, 125°10’56’E; Fig. 1). The station is located in a shelf area with a water depth of approximately 50 m. Despite the long distance (276 km) from the nearest land, daily observations are possible because the station has residential facilities. Water sampling and observation of environmental parameters were performed on the deck every morning from 25 March to 19 May, 2014. Seawater was sampled from the surface using a 5-L Niskin bottle.

### Prokaryotic abundances and tritiated thymidine incorporation rates

Samples for determining prokaryotic abundances were preserved in a mixture of paraformaldehyde and glutaraldehyde (final concentration of 1% and 0.05%, respectively) (Marie, Vaulot and Partensky 1996) and frozen at −20°C. Prokaryotic cells were counted by flow cytometry after staining with SYBR Green I (Sigma-Aldrich, St. Louis, MO, USA) (Marie *et al*. 1997). Tritiated thymidine ([^3^H-methy]thymidine) incorporation rates (TTI) were measured using methods described in a previous study (Choi *et al*. 2005). Duplicate 10-mL samples and a formalin-killed blank were incubated for approximately 1 h. The subsequent procedures were conducted as described previously (Choi *et al*. 2005).

### DNA extraction, PCR amplification and MiSeq sequencing

Prokaryotic community composition was analysed by high-throughput amplicon sequencing using the MiSeq platform. For DNA analysis, 1 or 2 L of seawater was filtered through a 0.2-μm Supor^®^ filter (47 mm diameter, Gelman Sciences, Ann Arbor, MI, USA). The filters soaked with 1 mL of STE buffer (100 mM NaCl, 10 mM Tris-HCl, 1 mM EDTA, pH 8.0) were stored at −20°C in the field and then at −70°C in the laboratory. DNA extraction and purification were conducted as previously described (Choi *et al*. 2016).

To amplify a V3-V4 hypervariable of prokaryotic 16S rRNA genes, a prokaryotic universal primer targeting both bacteria and archaea was used (Pro341F and Pro805; Takahashi *et al*. 2014). General procedures for PCR amplification, clean up and indexing PCR for MiSeq sequencing followed the instructions described in the MiSeq manual (Illumina 2013; Illumina, Inc., San Diego, CA, USA). FastStart Taq DNA polymerase (Roche Diagnostics, Basel, Switzerland) was used in the PCR reactions, and the annealing temperature of the first PCR reaction was 55°C. After clean-up of the second PCR reaction using AMPure XP beads (Beckman Coulter, Fullerton, CA, USA), final PCR products were quantified using a NanoDrop 1000 spectrophotometer (Thermo Fisher Scientific, Waltham, MA, USA). Identical quantities of each product were pooled, and the products were sequenced on the Illumina MiSeq 2 × 300 PE platform by ChunLab, Inc. (Seoul, Korea).

### Sequence data analysis

Reads from the MiSeq sequencing were analysed using the program mothur (Schloss *et al*. 2009) as suggested by the MiSeq standard operating procedures (http://www.mothur.org/wiki/MiSeq_SOP) (Kozich *et al*. 2013). After assembling paired-end sequences, contigs with ambiguous bases (N) over two, shorter than 400 bp or longer than 525 bp, and homopolymers greater than nine were removed. The EzBioCloud 16S database, which has detailed species-level taxonomic data, was used as a reference database for alignment and classification in the mothur program (http://www.ezbiocloud.net/resources/pipelines). After alignment, sequences that were likely due to PCR errors were removed using the ‘pre.cluster’ command, which permits up to four differences between sequences; then, chimeric sequences were also removed using the ‘chimera.uchime’ command. Sequence subsampling was performed to normalise the number of sequences per sample. The remaining sequences were assigned to operational taxonomic units (OTUs) using a 97% sequence identity threshold. Each OTU was classified using the naïve Bayesian classifier with a bootstrap cut-off of 80% (Wang *et al*. 2007). α-Diversity indices were calculated using the mothur program. The number of obtained reads per sample varied markedly from 705 and 102 485 in this study. Thus, before the analyses of composition and diversity. The number of reads of each sample was normalised to 2471 reads, although the OTU numbers tended to be undersaturated (Good's coverage range between 0.89 and 0.98) at the sequencing depth (Fig. S1, Supporting Information). As the read number for four samples was less than 2471, these samples were excluded from the diversity analysis. All sequence reads have been submitted to the National Center for Biotechnology Information sequence read archive (http://www.ncbi.nlm.nih.gov/Traces/sra; accession number SRX3267927).

### Oceanographic analyses

Chl *a*, extracted in 90% (v/v) acetone, was measured using a Turner fluorometer (10AU; Turner Designs, Sunnyvale, CA, USA) (Parsons, Maita and Lalli 1984). Size-fractionated chl *a* was measured by passing filtrates through a 20-μm nylon mesh and 3-μm polycarbonate membrane, respectively, on a GF/F membrane (Whatman, GE Healthcare, Little Chalfont, UK). Seawater temperature and salinity were measured using the Castaway CTD profiler (SonTek, San Diego, CA, USA).

## RESULTS

### Physical characteristics of the study area

**Figure 2.**
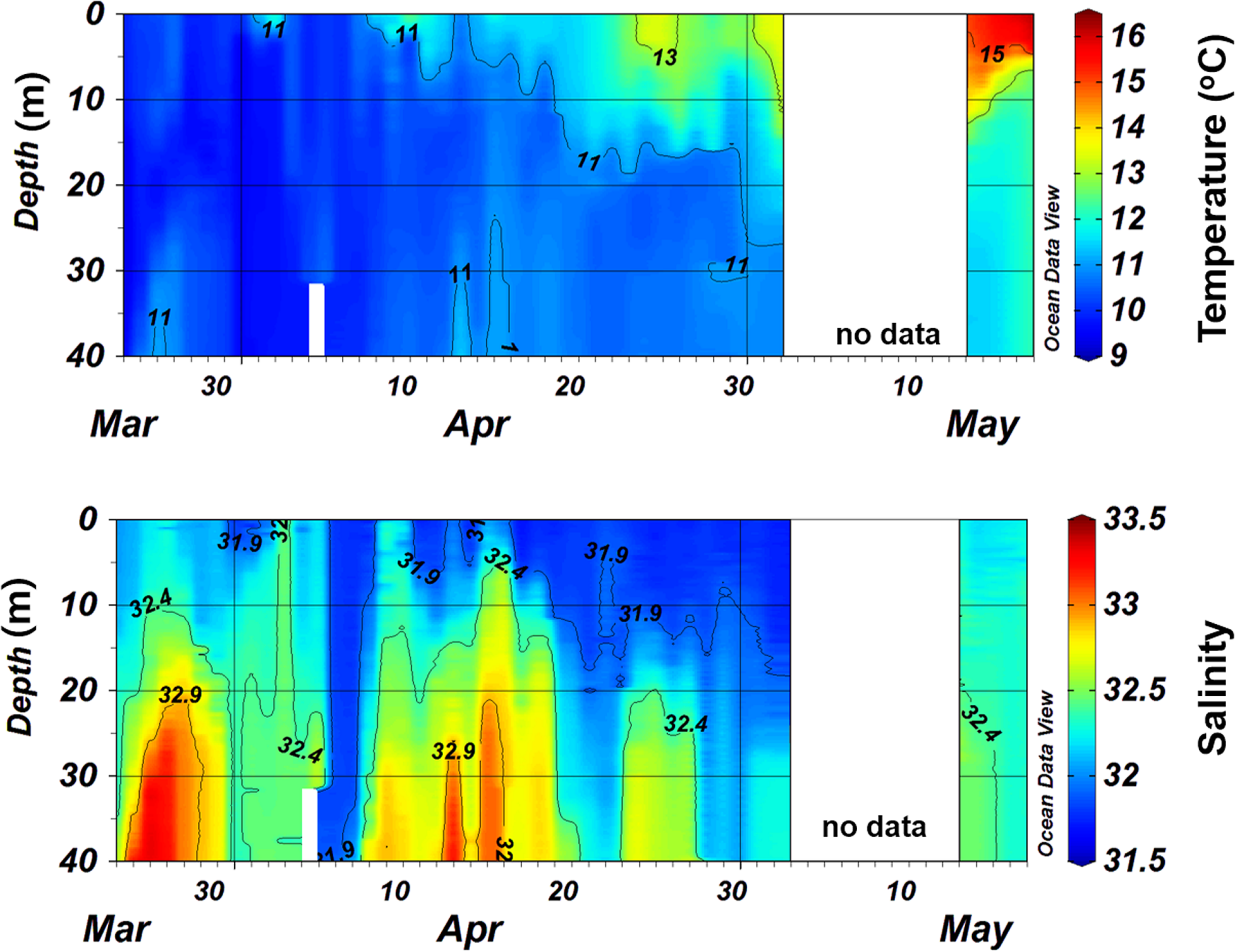
Time-series changes in seawater temperature and salinity measured over the study period. Due to the loss of the CTD profiler, no data were available for early May.

Seawater temperature tended to increase over the study period (Fig. 2). Thermal stratification began to develop in mid-April. Subsequently, the stratification was intensified due to surface warming by increased solar radiation; thus, seawater temperature increased from 9.9°C to 16.1°C at the end of the study. Salinity showed a pattern of slight decrease within a narrow range between 30.7 and 32.5 during the study (Fig. 2).

### Chl *a*, prokaryotic abundance and production

**Figure 3.**
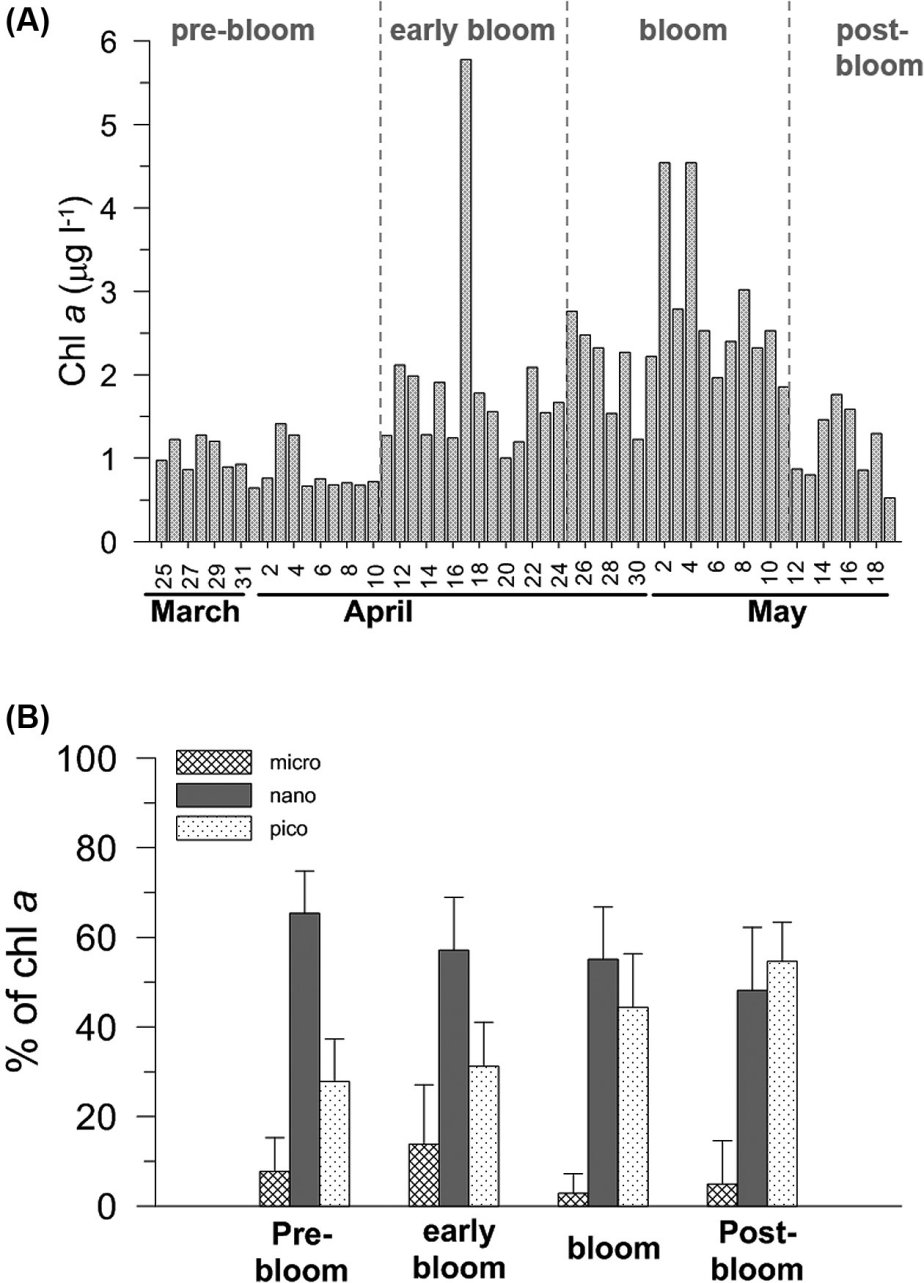
Changes in (**A**) chlorophyll *a* (chl *a*) concentrations and (**B**) percentages of chl *a* in each size fraction during the study. Vertical bars and error bars represent means and standard deviations, respectively.

Chl *a* ranged from 0.5 to 5.8 μg L^−1^, showing a 10 × variation over the study period (Fig. 3A). In mid-April, chl *a* was approximately doubled, and there was an abrupt one-day increase of chl *a* up to 5.8 μg L^−1^, principally due to diatom (mainly *Skeletonema costatum*, unpublished data) on 17 April. Similarly, chl a spikes were observed on May 2 and 4. An intense algal bloom began in late April and a relatively high chl *a* concentration of more than 2 μg L^−1^ was maintained for approximately 10 days. Thereafter, chl *a* gradually decreased. During the study period, chl *a* in the nano-sized fraction (3−20 μm) was most dominant, but tended to decrease slightly (Fig. 3B). Conversely, pico-fraction chl *a* (less than 3 μm) was relatively low in late-March but gradually increased to become the most dominant in the post-bloom period. Thus, both pico- and nano-sized phytoplankton were the most dominant algal groups in the spring bloom in the shelf waters of the East China Sea.

**Figure 4.**
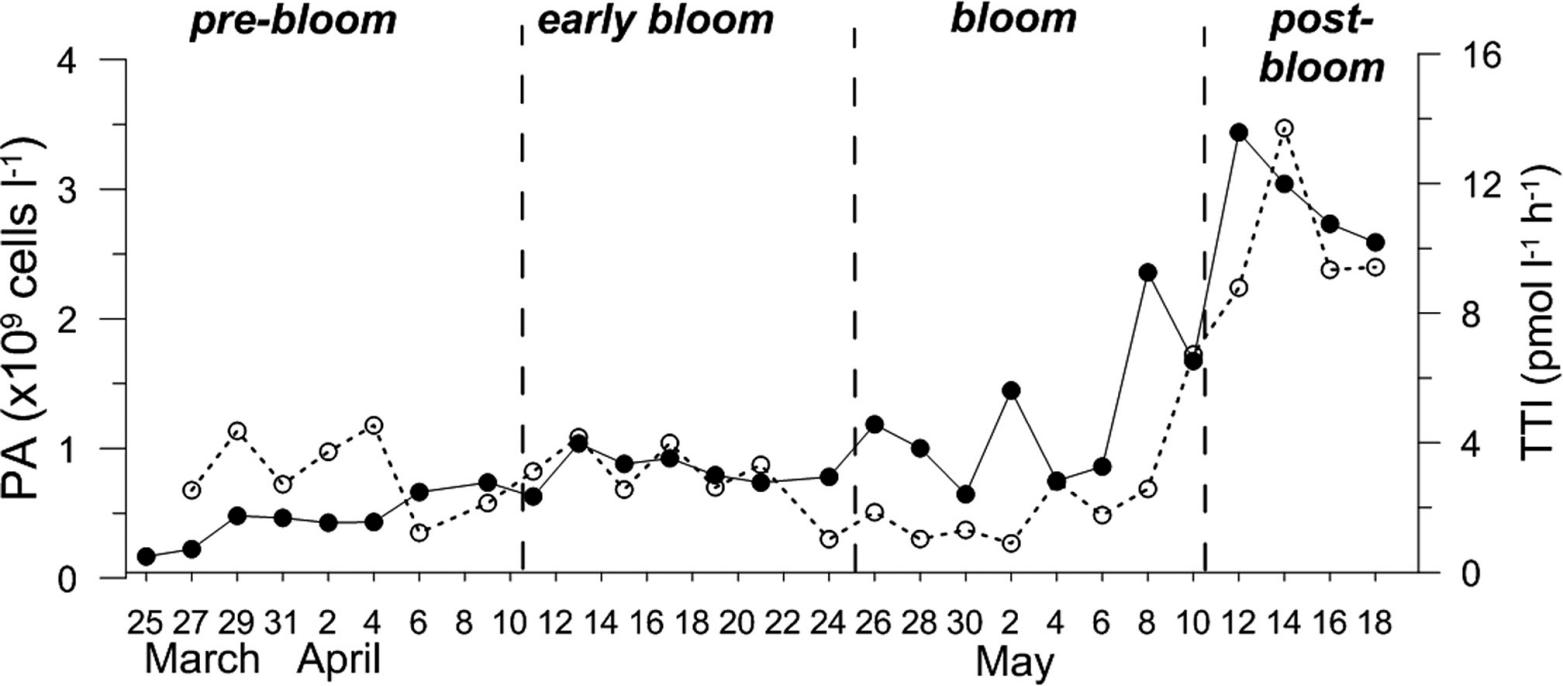
Changes in prokaryotic abundances (PA; closed circles) and production (TTI: tritiated thymidine incorporation rates; open circles) during the study.

Prokaryotic abundance and TTI tended to be affected directly by the spring algal bloom (Fig. 4). The abundance and TTI fluctuated at relatively low levels up to the early stages of the bloom. However, beginning in the middle phase of the bloom, prokaryotes grew exponentially with about a one-week lag time after the algal bloom, showing a more than 10-fold increase.

### Prokaryotic composition changes

**Figure 5.**
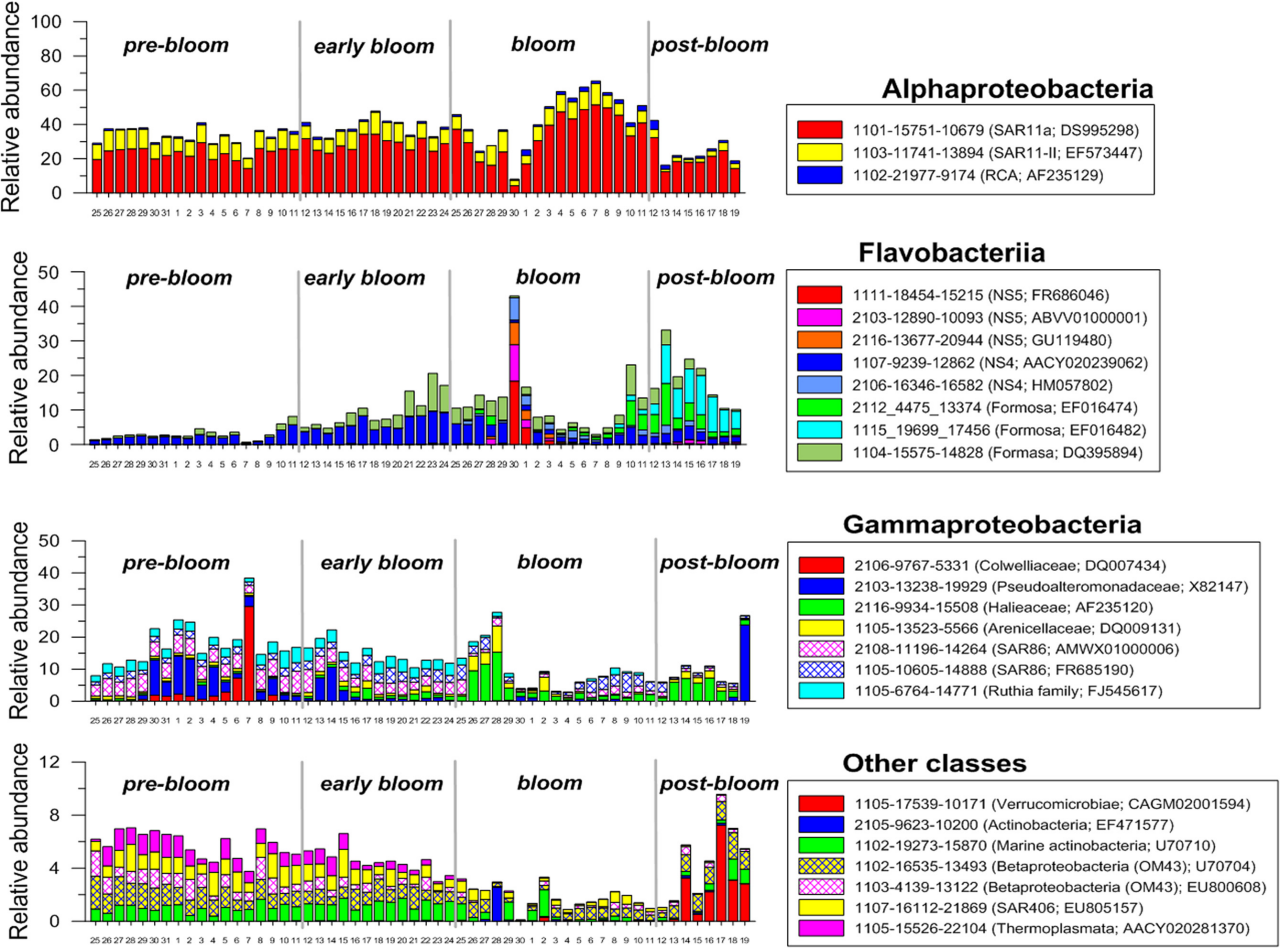
Changes in the relative abundance (%) of major prokaryotic OTUs during the study. The phylogenetic positions and temporal changes in other major OTUs can be seen in Figs S2–S5 (Supporting Information).

Most of the prokaryotic groups showed a successional change in relative abundance among groups for approximately two months in the spring and the shift occurred rapidly over a time scale of a few days (Fig. 5; Figs S2–S6, Supporting Information).

*Alphaproteobacteria* was found to be most dominant prokaryotes throughout the study period (Fig. S2, Supporting Information). Especially, at the middle stage of the bloom, bacteria affiliated with the SAR11 clade showed a maximum dominance of up to 80% (Fig. 5; Fig. S3, Supporting Information). An OTU belonging to SAR11 of the *Alphaproteobacteria* (mainly classified as subgroup Ia containing *Candidatus* Pelagibacter ubique) occupied from 13% to 52% of prokaryotic sequences during the study period (Fig. 5; Fig. S3, Supporting Information). This OTU was the most dominant genotype at almost all time periods and, interestingly, increased in dominance during the algal bloom. However, other genotypes belonging to the SAR11 cluster II tended to decrease after the algal bloom. The *Roseobacter* clade was the second most dominant of the *Alphaproteobacteria*, and diverse genotypes appeared over the study period compared with the SAR11 group (Fig. S3, Supporting Information). Further, temporal distribution of the genotypes was variable during the bloom period. *Amylibacter* species belonging to the NAC11-7 clade increased after the algal bloom, while a genotype closely affiliated with *Ascidiaceihabitans* species (AF235129) increased in relative proportion during the bloom (Fig. S3, Supporting Information). By contrast, genotypes closely related to *Sulfitobacter* showed their maxima during the early bloom period. OTUs related to *Puniceispirillum* and OM38 did not show significant changes during the study (Fig. S3, Supporting Information).

*Gammaproteobacteria* was the second dominant prokaryotes and their composition varied greatly during the study period (Fig. S2, Supporting Information). At the beginning of the bloom, as chl *a* increased to over 3 μg L^−1^, two OTUs (2116-9934-15508 and 1105-13523-5566) affiliated with *Luminiphilus* and *Arenicellaceae* species in the class *Gammaproteobacteria* became the dominant species for three days and then decreased (Fig. 5). At the post-bloom phase, *Luminiphilus* species ( 2116-9934-15508) appeared again at up to 8% (Fig. 5). Many other gammaproteobacterial groups appeared during the spring (Fig. 5; Fig. S4, Supporting Information), but their temporal dominance varied among genotypes. SAR86 and Ruthia groups were consistently found and predominated during the period before the algal bloom. Other OTUs belonging to the families *Colwelliaceae*, *Pseudoalteromonadaceae* and *Alteromonadaceae* were occasionally dominant in the pre-bloom period.

Relative dominance of bacteria belonging to the class *Flavobacteriia* was low at the pre-bloom period. However, they increased gradually after early bloom phase (Fig. S2, Supporting Information). A variety of *Flavobacteriia* groups appeared around the spring bloom period (Fig. 5; Fig. S5, Supporting Information). Many of them began to appear during the bloom period in particular. Among them, the NS4, NS5 and Formosa clades were the most dominant *Flavobacteriia*. Two OTUs in NS4 and most of the OTUs in the NS5 clade were dominant at the early bloom stage (over 40% of total sequences) and then tended to decrease during the bloom and post-bloom periods. However, in the initial post-bloom phase with an apparent drop in chl *a* to less than 2 μg L^−1^, other bacteria in the NS4 clade and *Formosa* species became dominant up to over 10%.

The other prokaryotic classes accounted for only minor fractions with different temporal patterns. *Roseibacillus* species belonging to *Verrucomicrobiae* appeared to occupy up to 8% of the total reads in the post-bloom period (Fig. 5; Fig. S6, Supporting Information). *Betaproteobacteria* showed a tendency to decrease during the bloom, but were present during the study period. *Deltaproteobacteria*, *Marinimicrobia* (SAR406) and two archaeal groups, *Thermoplasmata* and *Thaumarchaeota*, gradually decreased over time and nearly disappeared during the bloom and post-bloom period (Fig. 5; Fig. S6, Supporting Information).

### Prokaryotic community diversity during algal blooms

**Figure 6.**
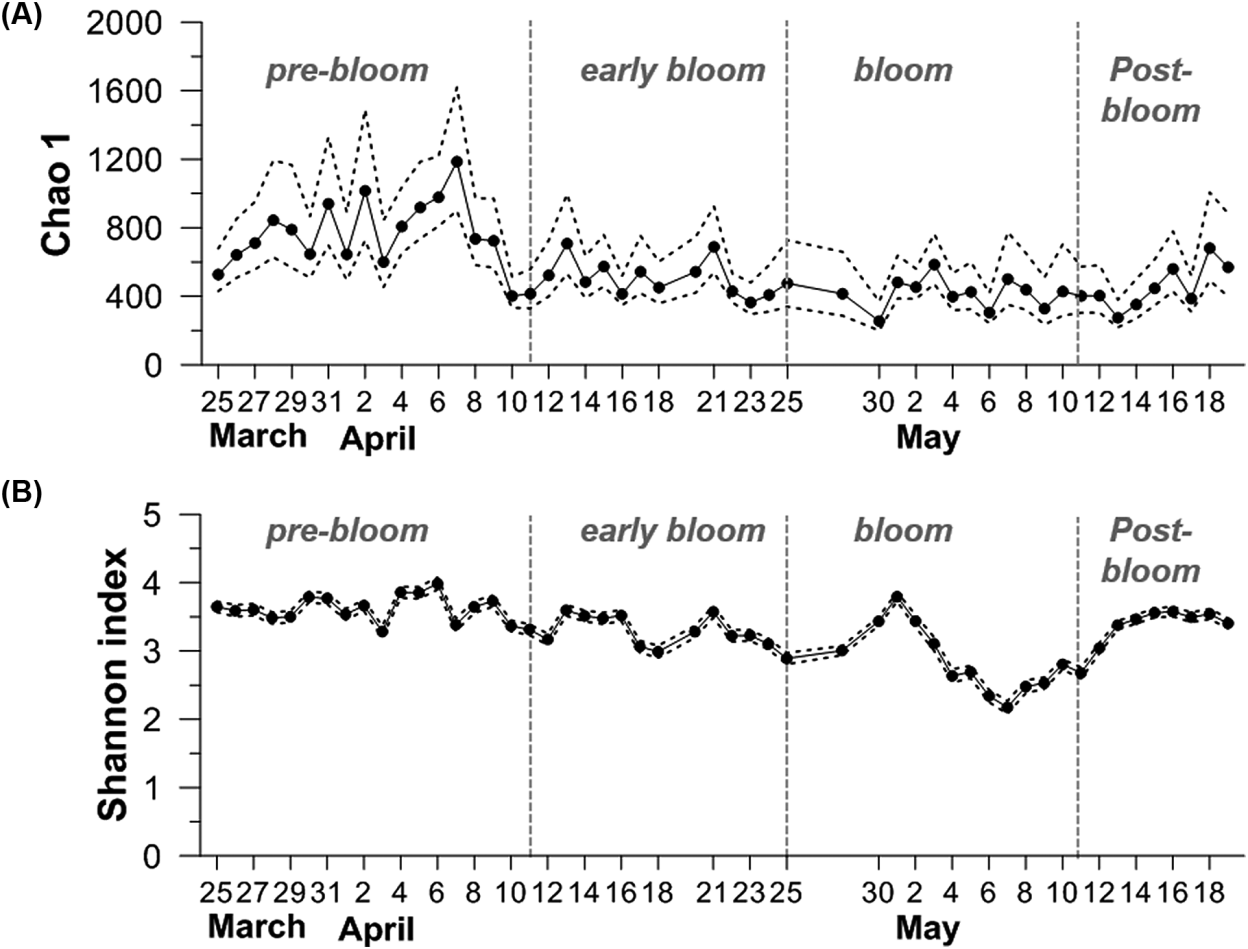
Changes in prokaryotic species richness and diversity using the Chao1 estimator and Shannon diversity index, respectively. Dotted lines represent 95% confidence intervals.

During the spring, prokaryotic species richness and diversity estimated by Chao1 and Shannon's diversity index (H’), respectively, tended to decrease from the early spring until the bloom period (Fig. 6). The Chao1 indexes in the bloom period were about two times lower than those in the pre-bloom period. However, the species richness tended to rebound during the post-bloom period. During the bloom, the H’ decreased rapidly after a gradual increase at the beginning of the bloom, and then recovered after the late bloom.

## DISCUSSION

Finely resolved prokaryotic responses to a spring algal bloom in offshore shelf waters were elucidated by daily observations for approximately two months on the IORS. The general responses seemed similar to those observed in coastal areas in previous studies. However, daily shifts in diverse bacterial genotypes (species and subspecies level) during and after the bloom were revealed by analysing daily samples using a high-throughput sequencing approach.

A typical spring bloom in coastal waters, which is developed by the growth of relatively large phytoplankton such as diatoms and dinoflagellates, shows very high chl *a* concentration with a scale of tens μg L^−1^ during the bloom (Cloern 1996; Fandino *et al*. 2001; Larsen *et al*. 2004; Tan *et al*. 2015; Yang *et al*. 2015; Bunse *et al*. 2016; Needham and Fuhrman 2016; Teeling *et al*. 2016). However, the magnitude of the spring bloom in offshore shelf waters in this study was relatively small (chl *a* less than 5 μg L^−1^) compared with those in coastal areas. Furthermore, the dominant phytoplankton during the bloom period were not large, but nano- and pico-phytoplankton. This bloom situation in offshore waters elicited a different prokaryotic response than that observed in coastal spring algal blooms. Prokaryotic abundances and production changed dynamically, and prokaryotic composition also showed a successional shift in the offshore shelf waters in the spring. The changes in prokaryotic parameters were particularly dramatic before and after the bloom. The temporal shift of dominant taxa at a species level occurred within a few days.

### Prokaryotic diversity during the bloom

The algal bloom simplified prokaryotic composition in terms of both species richness and diversity in offshore shelf waters (Fig. 6). Prokaryotic diversity in particular sharply decreased during the bloom phase. Considering the rapid increases in prokaryotic abundance and production during the bloom period (up to 2-fold and 10-fold, respectively; Fig. 4), the lower diversity during the bloom seemed to be due to the rapid growth of a relatively small number of prokaryotic species that rapidly adapted to the enriched DOM conditions during the bloom. Consistently, a study conducted in the southern North Sea in the spring showed a low richness and reduced Shannon indices in samples from the bloom area (Wemheuer *et al*. 2014). Similarly, in a coastal area of the Xiamen Sea, species richness was lower at the bloom station, although the Shannon index did not show a significant difference (Yang *et al*. 2015). However, both species richness and diversity tended to gradually increase as the bloom ended, suggesting that prokaryotic diversity was rapidly recovered, accompanied with an obvious change in prokaryotic composition.

### Responses of prokaryotic composition during the algal bloom

The general picture of bacterial succession during the bloom was similar to previous studies. In previous time-series studies conducted in coastal areas, *Alphaproteobacteria*, including those of *Roseobacter* clade-affiliated (RCA) lineage and several groups belonging to *Gammaproteobacteria* and *Flavobacteriia*, were revealed as bloom-associated prokaryotic taxa, which can rapidly utilise various organic matter produced by phytoplankton blooms (Teeling *et al*. 2012; Buchan *et al*. 2014; Needham and Fuhrman 2016; Teeling *et al*. 2016).

However, the day-to-day change in prokaryotic composition at the species level differentiated our study from the previous results in some respects. The Ia subgroup of SAR11 was found to be the most dominant bacteria during the bloom phase in this study. Given that the increase in this bacterial group occurred concurrently with the initial increase in prokaryotic abundance and production (Fig. 4), this subgroup must rapidly utilise fresh and labile DOM produced by the algal bloom, and thus exhibited rapid growth. In a time-series study conducted at an offshore station (Needham and Fuhrman 2016), when the primary bloom (mainly diatoms) occurred with a maximum chl *a* up to 10 μg L^−1^, the relative percentage of SAR11 decreased to be surpassed by *Bacteroidetes* during the bloom, even though the Ia subgroup of SAR11 was dominant in non-bloom conditions. However, during a subsequent bloom by smaller flagellates and picophytoplankton belonging to prymnesiophytes and *Ostreococcus*, subgroup Ia tended to increase along with chl *a*. In our study, prymnesiophytes, which are closely related to *Chrysochromulina*, were dominant exclusively during the algal bloom (unpublished data). Thus, the increase in the Ia subgroup in this study is consistent with the previous study, suggesting that phytoplankton composition is of primary importance in determining the bacterial composition during spring blooms. Therefore, a close interaction between phytoplankton and prokaryotic groups may explain the diverse responses observed during algal blooms (Tan *et al*. 2015; Bunse *et al*. 2016; Needham and Fuhrman 2016). Similarly, a study addressing differences in dominant bacterial species between diatom- and flagellate-dominated blooms using microcosm experiments has also been reported (Pinhassi *et al*. 2004). Interestingly, spikes in chl *a* were found on April 17, May 2 and May 4, probably due to advection of bloom water from another location rather than *in situ* growth. On April 17, the increase in chl *a* was due to an increase in *Skeletonema* diatom. However, the prokaryotic compositions on these days were similar to those on adjacent days (Fig. 5). Thus, short-term algal blooming could not have led to the subsequent changes in the prokaryotic community, and the time-lag between phytoplankton and prokaryotic blooms is a plausible explanation for this uncoupling.

In a previous time-series monitoring of the prokaryotic community during an algal bloom conducted in coastal waters (Teeling *et al*. 2012), the NAC11-7 and RCA lineages dominated during the early and late bloom, respectively. In addition, *Ulvibacter*, *Formosa-*related and *Polaribacter* species in the *Flavobacteriia*, and SAR92 and *Reinekea* species in the *Gammaproteobacteria*, increased successively to become dominant taxa at the late bloom and post-bloom stages. In another time-series study conducted in an offshore station, Formosa-related lineage, *Polaribacter*, and *Verrucomicrobium* and SAR92 taxa became most abundant in particle-attached or free-living fractions (Needham and Fuhrman 2016). Likewise, in the offshore shelf waters of the East China Sea, we observed a slight increase in RCA lineages in the bloom and post-bloom periods, and dominance of *Formosa-*related species in the post-bloom period. However, we did not observe significant levels of *Ulvibacter*, *Polaribacter*, *Reinekea* or SAR92 species during that time period.

The response of archaea to spring algal blooms has not yet been well-studied. Interestingly, in this study, taxa affiliated with *Thermoplasmata* of the *Euryarchaeota* and ammonium-oxidising *Thaumarchaeota* were found at significant levels in early spring, composing up to 3% and 1% of total prokaryote reads, respectively (Fig. 5; Fig. S6, Supporting Information). However, they tended to decrease as chl *a* increased, and became nearly insignificant during and after the spring bloom, likely due to competition by bacteria and phytoplankton for organic matter and inorganic nutrients, such as ammonia, under the bloom conditions. Similar results were also obtained in an offshore area of Southern California, where MGII euryarchaeal taxa composed up to 30% of the prokaryotic community before a bloom, but sharply dropped as the bloom began (Needham and Fuhrman 2016).

These shifts in prokaryotic composition during the bloom were likely affected by diverse factors, such as the quality and quantity of organic matter from primary production and subsequent prokaryotic decomposition, viral lysis, grazing and the supply of particulate organic matter from the senescence and death of phytoplankton (Smith *et al*. 1992; Biddanda and Benner 1997; Alonso-Saez and Gasol 2007; Parsons *et al*. 2012; Buchan *et al*. 2014; Tarran and Bruun 2015). Given the steep increase in prokaryotic abundance and production, substrate supply seems to be important in the growth of prokaryotes during algal blooms and the early post-bloom period. Thus, changes in genetic repertoires, including carbohydrate-active enzymes and transporters, may be important for the utilisation of algal substrates and successive growth of specific bacterial taxa during these periods (Teeling *et al*. 2016). Indeed, there was a time-delayed correlation between the dominant phytoplankton taxa and specific prokaryotes during the spring bloom (Fig. S7, Supporting Information), suggesting that changes in phytoplankton taxa might be a primary factor regulating the responses of bacterial composition to algal blooms. However, the temperature increase as well as top-down factors, such as grazing and viral lysis, which were not estimated in this study, cannot be neglected as significant factors regulating changes in composition during a bloom (Yager *et al*. 2001; Kataoka *et al*. 2009; Löder *et al*. 2011). In fact, temperature was a significant environmental factor explaining the variance of major prokaryotic taxa in both the CCA and network analyses in this study (Figs S7 and S8, Supporting Information).

In conclusion, daily observations at the ocean platform established in offshore waters of the East China Sea elucidated fine-scale changes in prokaryotic diversity and composition during the spring bloom. General trends in compositional shift, particularly at the class level, were similar to those observed in coastal areas. However, the responses of bloom-associated taxa at the species level rapidly shifted over a time scale of only a few days, likely due to dynamic changes in substrate conditions.

## Supplementary Material

Supplementary Data

## References

[bib1] Alonso-Saez L, Gasol JM Seasonal variations in the contributions of different bacterial groups to the uptake of low-molecular-weight compounds in northwestern Mediterranean coastal waters. Appl Environ Microbiol. 2007;73:3528–35. 17400772 10.1128/AEM.02627-06 PMC1932672

[bib2] Becker JW, Berube PM, Follett CL Closely related phytoplankton species produce similar suites of dissolved organic matter. Front Microbiol. 2014;5:111. 24748874 10.3389/fmicb.2014.00111 PMC3975126

[bib3] Biddanda B, Benner R Carbon, nitrogen, and carbohydrate fluxes during the production of particulate and dissolved organic matter by marine phytoplankton. Limnol Oceanogr. 1997;42:506–18.

[bib4] Billen G, Fontigny A Dynamics of a Phaeocystis-dominated spring bloom in Belgian coastal waters. 2. Bacterioplankton dynamics. Mar Ecol Prog Ser. 1987;37:249–57.

[bib5] Buchan A, LeCleir GR, Gulvik CA Master recyclers: features and functions of bacteria associated with phytoplankton blooms. Nat Revi Microbiol. 2014;12:686–98. 10.1038/nrmicro3326 25134618

[bib6] Bunse C, Bertos-Fortis M, Sassenhagen I Spatio-temporal interdependence of bacteria and phytoplankton during a baltic sea spring bloom. Front Microbiol. 2016;7:517. 27148206 10.3389/fmicb.2016.00517 PMC4838809

[bib7] Castberg T, Larsen A, Sandaa RA Microbial population dynamics and diversity during a bloom of the marine coccolithophorid Emiliania huxleyi (Haptophyta). Mar Ecol-Prog Ser. 2001;221:39–46.

[bib8] Cho BC, Choi JK, Chung CS Uncoupling of bacteria and phytoplankton during a spring diatom bloom in the mouth of the Yellow Sea. Mar Ecol Prog Ser. 1994;115:181–90.

[bib10] Choi DH, An SM, Chun S Dynamic changes in the composition of photosynthetic picoeukaryotes in the northwestern Pacific Ocean revealed by high-throughput tag sequencing of plastid 16S rRNA genes. Fems Microbiol Ecol. 2016;92:10.1093/femsec/fiv170. 10.1093/femsec/fiv170 26712350

[bib9] Choi DH, Yang SR, Hong GH Different interrelationships among phytoplankton, bacterial and environmental variables in dumping and reference areas in the East Sea. Aquat Microb Ecol. 2005;41:171–80.

[bib11] Cloern JE Phytoplankton bloom dynamics in coastal ecosystems: a review with some general lessons from sustained investigation of San Francisco Bay, California. Rev Geophys. 1996;34:127–68.

[bib12] Cole JJ, Findlay S, Pace ML Bacterial production in fresh and saltwater ecosystems: a cross-system overview. Mar Ecol Prog Ser. 1988;43:1–10.

[bib14] Ducklow HW, Kirchman DL, Quinby HL Stocks and dynamics of Bacterioplankton carbon during the spring bloom in the Eastern North-Atlantic ocean. Deep-Sea Res Pt II. 1993;40:245–63.

[bib13] Ducklow HW, Kirchman DL Bacterial dynamics and distribution during a spring diatom bloom in the Hudson River plume, USA. J Plankton Res. 1983;5:333–55.

[bib15] Fandino LB, Riemann L, Steward GF Variations in bacterial community structure duringa dinoflagellate bloom analyzed by DGGE and 16S rDNA sequencing. Aquat Microb Ecol. 2001;23:119–30.

[bib1_859_1531899570158] Illumina 16S Metagenomic Sequencing Library Preparation. 2013, https://support.illumina.com/downloads/16s_metagenomic_sequencing_library_preparation.html (18 July 2018, date last accessed).

[bib16] Kataoka T, Hodoki Y, Suzuki K Tempo-spatial patterns of bacterial community composition in the western North Pacific Ocean. J Marine Syst. 2009;77:197–207.

[bib17] Kerkhof LJ, Voytek MA, Sherrell RM Variability in bacterial community structure during upwelling in the coastal ocean. Hydrobiologia. 1999;401:139–48.

[bib18] Kozich JJ, Westcott SL, Baxter NT Development of a dual-index sequencing strategy and curation pipeline for analyzing amplicon sequence data on the MiSeq Illumina sequencing platform. Appl Environ Microbiol. 2013;79:5112–20. 23793624 10.1128/AEM.01043-13 PMC3753973

[bib19] Kristiansen S, Farbrot T, Naustvoll L-J Spring bloom nutrient dynamics in the Oslofjord. Mar Ecol Prog Ser. 2001;219:41–49.

[bib20] Larsen A, Flaten GAF, Sandaa RA Spring phytoplankton bloom dynamics in Norwegian coastal waters: microbial community succession and diversity. Limnol Oceanogr. 2004;49:180–90.

[bib21] Löder MGJ, Meunier C, Wiltshire KH The role of ciliates, heterotrophic dinoflagellates and copepods in structuring spring plankton communities at Helgoland Roads, North Sea. Mar Biol. 2011;158:1551–80.

[bib23] Marie D, Partensky F, Jacquet S Enumeration and cell cycle analysis of natural populations of marine picoplankton by flow cytometry using the nucleic acid stain SYBR Green I. Appl Environ Microbiol. 1997;63:186–93. 16535483 10.1128/aem.63.1.186-193.1997 PMC1389098

[bib22] Marie D, Vaulot D, Partensky F Application of the novel nucleic acid dyes YOYO-1, YO-PRO-1, and PicoGreen for flow cytometric analysis of marine prokaryotes. Appl Environ Microbiol. 1996;62:1649–55. 8633863 10.1128/aem.62.5.1649-1655.1996 PMC167939

[bib24] Maynard NG, Clark DK Satellite color observations of spring blooming in Bering Sea shelf waters during the ice edge retreat in 1980. J Geophys Res Oceans. 1987;92:7127–39.

[bib25] Meon B, Kirchman DL Dynamics and molecular composition of dissolved organic material during experimental phytoplankton blooms. Mar Chem. 2001;75:185–99.

[bib26] Myklestad SM Release of extracellular products by phytoplankton with special emphasis on polysaccharides. Sci Total Environ. 1995;165:155–64.

[bib27] Needham DM, Fuhrman JA Pronounced daily succession of phytoplankton, archaea and bacteria following a spring bloom. Nat Microbiol. 2016;1:16005. 27572439 10.1038/nmicrobiol.2016.5

[bib28] Park KA, Kang CK, Kim KR Role of sea ice on satellite-observed chlorophyll-a concentration variations during spring bloom in the East/Japan sea. Deep-Sea Res Pt II. 2014;83:34–44.

[bib29] Parsons RJ, Breitbart M, Lomas MW Ocean time-series reveals recurring seasonal patterns of virioplankton dynamics in the northwestern Sargasso Sea. ISME J. 2012;6:273–84. 21833038 10.1038/ismej.2011.101 PMC3260494

[bib30] Parsons TR, Maita Y, Lalli CM A Manual of Chemical and Biological Methods for Seawater Analysis, Pergamon Amsterdam 1984.

[bib31] Pinhassi J, Sala MM, Havskum H Changes in bacterioplankton composition under different phytoplankton regimens. Appl Environ Microbiol. 2004;70:6753–66. 15528542 10.1128/AEM.70.11.6753-6766.2004 PMC525254

[bib32] Riemann L, Steward GF, Azam F Dynamics of bacterial community composition and activity during a mesocosm diatom bloom. Appl Environ Microbiol. 2000;66:578–87. 10653721 10.1128/aem.66.2.578-587.2000 PMC91866

[bib33] Romera-Castillo C, Sarmento H, Álvarez-Salgado XA Production of chromophoric dissolved organic matter by marine phytoplankton. Limnol Oceanogr. 2010;55:446–54.

[bib34] Schloss PD, Westcott SL, Ryabin T Introducing mothur: open-source, platform-independent, community-supported software for describing and comparing microbial communities. Appl Environ Microbiol. 2009;75:7537–41. 19801464 10.1128/AEM.01541-09 PMC2786419

[bib35] Sison-Mangus MP, Jiang S, Kudela RM Phytoplankton-associated bacterial community composition and succession during toxic diatom bloom and non-bloom events. Front Microbiol. 2016;7:1433. 27672385 10.3389/fmicb.2016.01433 PMC5018474

[bib36] Smetacek V, Cloern JE Oceans - on phytoplankton trends. Science. 2008;319:1346–8. 18323440 10.1126/science.1151330

[bib37] Smith DC, Simon M, Alldredge AL Intense hydrolytic enzyme activity on marine aggregates and implications for rapid particle dissolution. Nature. 1992;359:139–42.

[bib38] Takahashi S, Tomita J, Nishioka K Development of a prokaryotic universal primer for simultaneous analysis of bacteria and archaea using next-generation sequencing. PLoS One. 2014;9:e105592. 25144201 10.1371/journal.pone.0105592 PMC4140814

[bib39] Tan SJ, Zhou J, Zhu XS An association network analysis among microeukaryotes and bacterioplankton reveals algal bloom dynamics. J Phycol. 2015;51:120–32. 26986263 10.1111/jpy.12259

[bib40] Tarran GA, Bruun JT Nanoplankton and picoplankton in the Western English Channel: abundance and seasonality from 2007–2013. Prog Oceanogr. 2015;137:446–55.

[bib41] Taylor JR, Ferrari R Shutdown of turbulent convection as a new criterion for the onset of spring phytoplankton blooms. Limnol Oceanogr. 2011;56:2293–307.

[bib43] Teeling H, Fuchs BM, Becher D Substrate-controlled succession of marine bacterioplankton populations induced by a phytoplankton bloom. Science. 2012;336:608–11. 22556258 10.1126/science.1218344

[bib42] Teeling H, Fuchs BM, Bennke CM Recurring patterns in bacterioplankton dynamics during coastal spring algae blooms. Elife. 2016;5:e11888. 27054497 10.7554/eLife.11888 PMC4829426

[bib44] Thomas AC, Townsend DW, Weatherbee R Satellite-measured phytoplankton variability in the Gulf of Maine. Cont Shelf Res. 2003;23:971–89.

[bib45] Wang Q, Garrity GM, Tiedje JM Naive Bayesian classifier for rapid assignment of rRNA sequences into the new bacterial taxonomy. Appl Environ Microbiol. 2007;73:5261–7. 17586664 10.1128/AEM.00062-07 PMC1950982

[bib46] Weisse T, Scheffel-Moser U Uncoupling the microbial loop: growth and grazing loss rates of bacteria and heterotrophic nanoflagellates in the North Atlantic. Mar Ecol Prog Ser. 1991;71:195–205.

[bib47] Wemheuer B, Gullert S, Billerbeck S Impact of a phytoplankton bloom on the diversity of the active bacterial community in the southern North Sea as revealed by metatranscriptomic approaches. Fems Microbiol Ecol. 2014;87:378–89. 24111503 10.1111/1574-6941.12230

[bib48] Wetz MS, Wheeler PA Release of dissolved organic matter by coastal diatoms. Limnol Oceanogr. 2007;52:798–807.

[bib49] Xing P, Hahnke RL, Unfried F Niches of two polysaccharide-degrading Polaribacter isolates from the North Sea during a spring diatom bloom. ISME J. 2015;9:1410–22. 25478683 10.1038/ismej.2014.225 PMC4438327

[bib50] Yager PL, Connelly TL, Mortazavi B Dynamic bacterial and viral response to an algal bloom at subzero temperatures. Limnol Oceanogr. 2001;46:790–801.

[bib51] Yang CY, Li Y, Zhou B Illumina sequencing-based analysis of free-living bacterial community dynamics during an Akashiwo sanguine bloom in Xiamen sea, China. Sci Rep. 2015;5:8476. 25684124 10.1038/srep08476 PMC4329561

