# Supplementary material for: Daily variation in the prokaryotic community during a spring bloom in shelf waters of the East China Sea
Source: FEMS Microbiol Ecol. 2018 Jul 13;94(9):fiy134. doi: 10.1093/femsec/fiy134 (PMC6061848; doi:10.1093/femsec/fiy134)
Supplement: Supplementary Data [file fiy134_supplemental_figures.docx]

Figure S1. Rarefaction curves showing coverage at the subsampling size (read no. of 2471) of this study.

Figure S2. Changes in prokaryotic composition at the class level during the study.

Figure S3. Heatmap showing changes in relative abundance (%) of alphaproteobacterial genotypes.


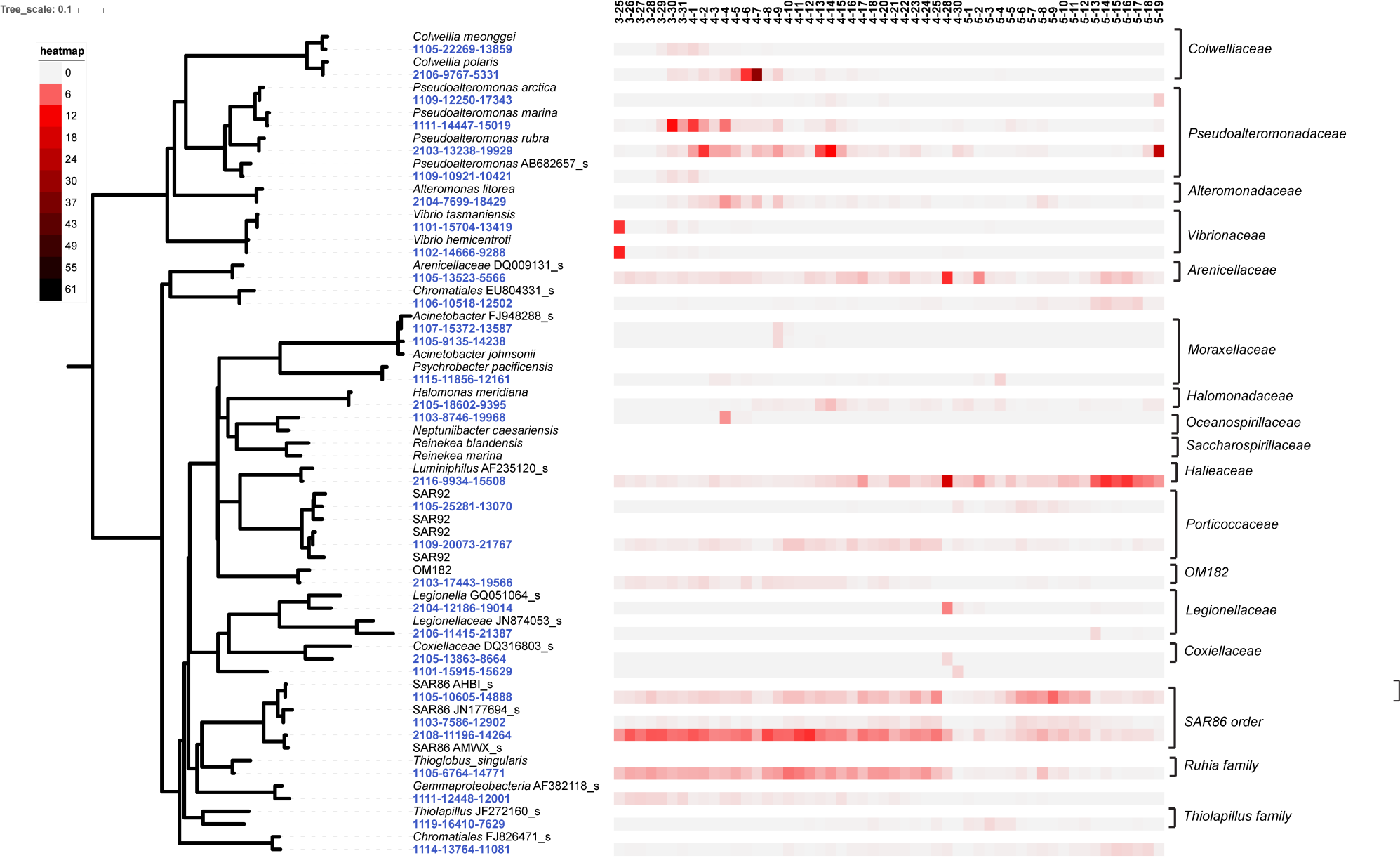


Figure S4. Heatmap showing changes in relative abundance (%) of gammaproteobacterial genotypes.


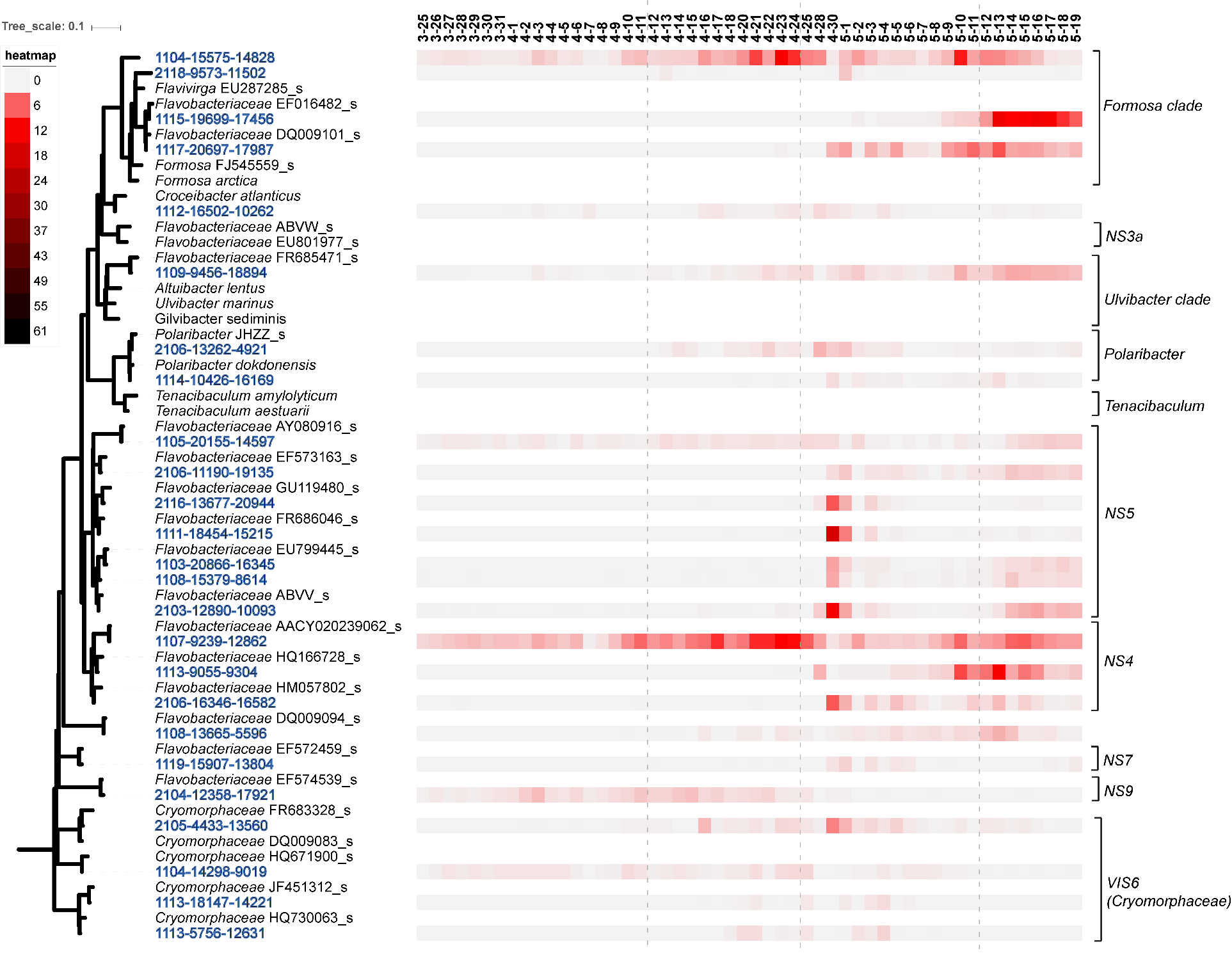


;Figure S5 Heatmap showing changes in relative abundance (%) of genotypes belonging to *Flavobacteriia*.


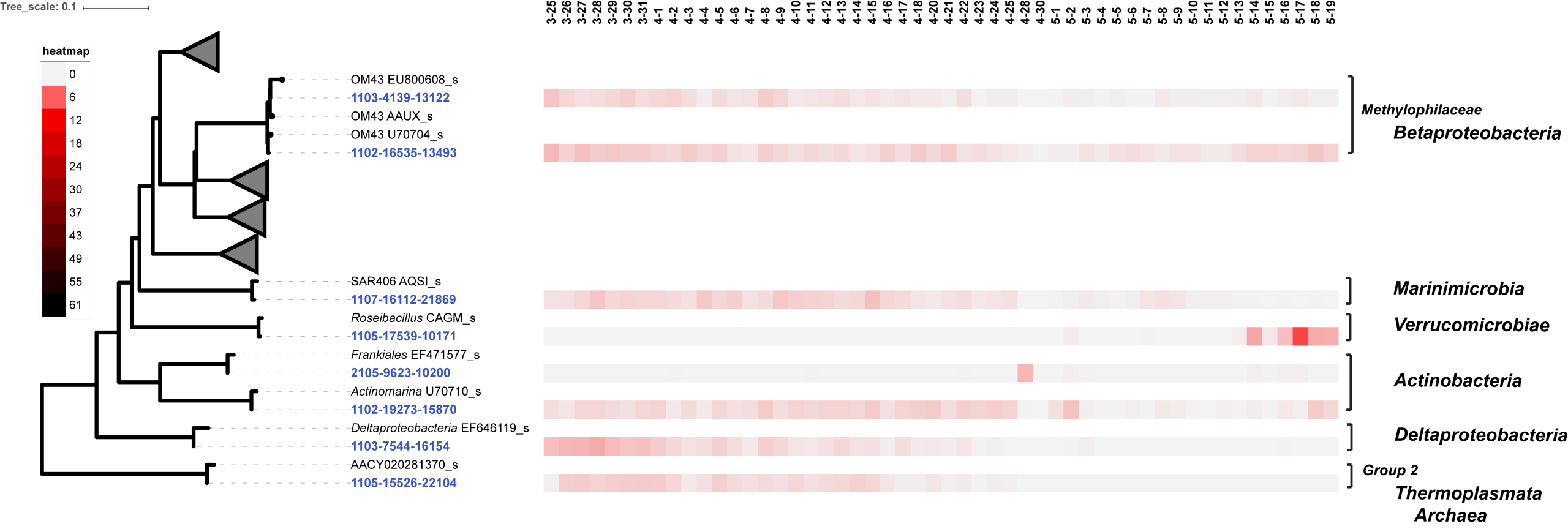


Figure S6. Heatmap showing changes in relative abundance (%) of minority genotypes


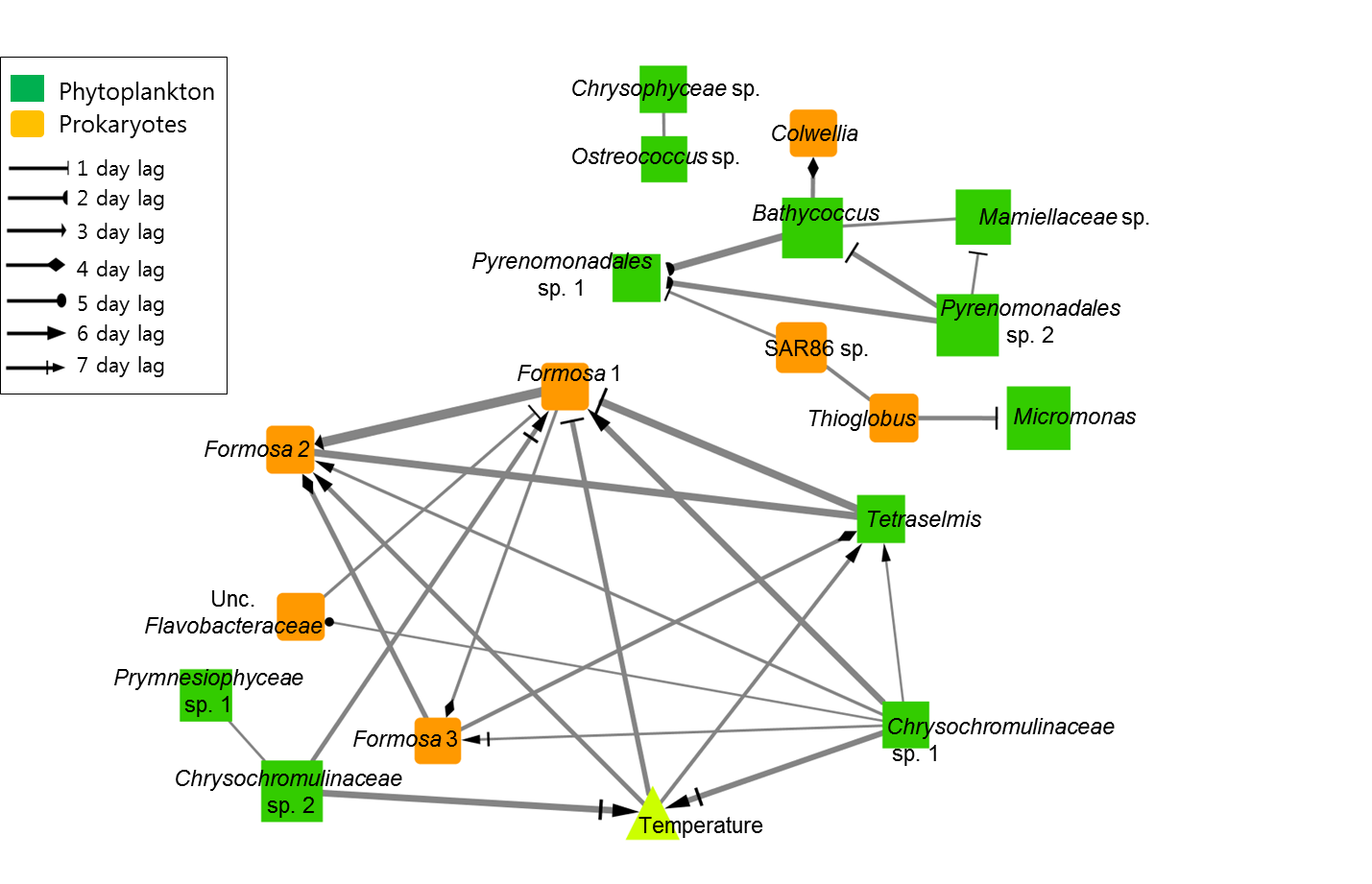


Figure S7. Microbial association network showing pairwise time-shifted Spearman’s correlations between phytoplankton and bacteria. Only edges with positive correlations (r > 0.80; p < 0.0001) between nodes and major OTUs occupying over 5% at least one sample were shown. The width of the lines represents correlation strength and the size of node symbols represents the average of relative OTU abundances during the study. Eukaryotic composition in whole seawater was analysed by plastid-16S rRNA gene sequencing using MiSeq platform (Choi et al., 2016. FEMS Microbiol. Ecol. 92:fiv170). Network analysis was conducted using the eLSA tool (http://meta.usc.edu/softs/lsa/).


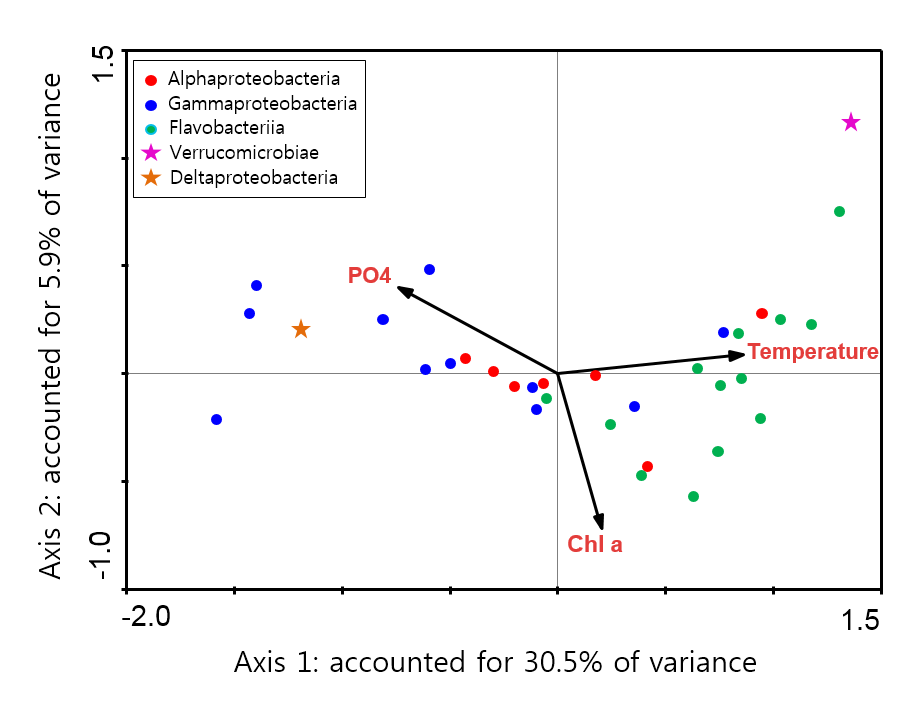


Figure S8. CCA (Canonical correspondence analysis) ordination plot showing the relationships between major prokaryotic OTUs and environmental parameters. CCA was performed using Canoco for Windows (ver. 4.51) to clarify the factors controlling major prokaryotic genotypes. Response variables (OTU %) were log(%+1)-transformed before the analysis, while explanatory variables (temperature, irradiance, nutrients [nitrate, phosphate and silicate] and chl a) were not transformed. The CCA model was produced with forward selection via Monte Carlo permutation significance tests (p < 0.05).
